# Cross-Sectional Associations Between Personal and General Views on Aging and Future Care Preparation in Individuals Aged 50 and Over and Living in the United Kingdom

**DOI:** 10.1177/07334648251398098

**Published:** 2025-11-20

**Authors:** Serena Sabatini, Fiona S. Rupprecht, Emily Mroz, Shelbie Turner, Robyn Capithorne Crainey, Eugene Y. H. Tang, Claire V. Burley, Jennifer Dunne, Elissa Burton, Stephanie Wong, Bethany Wilton-Harding, Tim Windsor, Rachel C. Ambagtsheer, Katya Numbers, Blossom Stephan

**Affiliations:** 1 School of Psychology, Faculty of Health and Medical Sciences, 3660 University of Surrey, Guildford, UK; 2 Department of Clinical Psychology and Psychobiology, University of Barcelona, Spain; 3 Department of Psychology, 26562 University of Kaiserslautern-Landau, Germany; 4 Nell Hodgson Woodruff School of Nursing, Emory University, Atlanta, GA, USA; 5 Division of Geriatrics and Palliative Medicine, Weill Cornell Medical College, New York, NY, USA; 6 Population Health Sciences Institute, 5994 Newcastle University, UK; 7 Dementia Centre of Excellence, Curtin enAble Institute, 1649 Curtin University, Perth, WA, Australia; 8 Faculty of Health Sciences, School of Allied Health, 1649 Curtin University, Perth, WA, Australia; 9 Brightwater Research Centre, Brightwater Care Group, Perth, WA, Australia; 10 Flinders Institute for Mental Health and Wellbeing, College of Education, Psychology and Social Work, 1065 Flinders University, Adelaide, SA, Australia; 11 Centre for Public Health Equity and Human Flourishing, Health and Education Vertical, 386703 Torrens University Australia, Adelaide, SA, Australia; 12 Centre for Healthy Brain Ageing (CHeBA), Discipline of Psychiatry & Mental Health, University of New South Wales, Sydney, SA, Australia

**Keywords:** awareness of age-related change, subjective aging, self-perceptions of aging, expectations regarding aging, essentialist beliefs about aging

## Abstract

**Introduction:**

Future care preparation refers to those processes during which individuals discuss with healthcare professionals or family members about their future care. This study investigated whether views on aging are associated with future care preparation and whether age moderates these associations.

**Methods:**

We conducted a cross-sectional survey with 1,699 participants (mean age = 68 years). Views on aging indicators were perceived age-related gains and losses and expectations regarding aging. Linear regressions were used.

**Results:**

Higher perceived age-related gains (B = 0.12; p-value <0.001) and losses (B = 0.16; p-value <0.001) and less positive expectations regarding physical health while aging (B = −0.11; p-value <0.05) were associated with higher future care preparation. Age did not moderate any association.

**Discussion:**

Given the importance of future care preparation in ensuring optimal health outcomes for older adults, encouraging older adults to have open and frank conversations about their views on aging may be an important first step in eliciting care needs.

**What This Paper Adds**

• This is the first study investigating the associations of views on aging with future care preparation.
• Study results suggest that both positive and negative personal experiences/views of aging may elicit future care preparation.
• Having some negative views about physical health in older age may make individuals wanting to plan for their future care.

**Applications of Study Findings**

• The promotion of more positive personal views on aging may foster future care preparation.
• Having conversations about their views on aging with family, friends, and/or healthcare professionals may be a first step that prones middle-aged and older individuals to think about their care needs.

## Introduction

The global proportion of older people (i.e., aged ≥65 years) is rapidly increasing. According to the United Nations, worldwide there are 830 million people aged ≥65 years, and this number is projected to reach 1.7 billion by 2054 (United Nations, 2024). In the United Kingdom it is estimated there are over 10 million people aged ≥65 years, making up 18% of its population (Centre for Ageing Better, 2023). To address this sociodemographic change, a public health priority is to expand health span alongside lifespan and to ensure adults have the resources that they need to navigate new health challenges (Bousquet et al., 2015).

### Future Care Preparation and its Benefits

*Future care preparation* refers to processes through which individuals equip themselves for the likelihood that they will increasingly rely on family members, close others, or healthcare professionals for support as they navigate new or persisting health challenges in late life. Future care preparation includes activities where individuals *become aware* of potential support needs, *gather information* about how to obtain the support they may need, *make decisions* about support they may or may not want, and eventually, take *concrete steps* to convey their preferences (Sörensen et al., 2017). While this type of preparation may include forms of *advance care planning,* such as completion of an advance directive or nomination of a health care surrogate to guide medical care, it also includes a range of other preparatory activities that are fit to the current and anticipated needs and knowledge of individuals. Examples of future care preparation activities are talking to family members or close others to reflect on whether one may need help or care in the future; making concrete plans about future care option by talking with healthcare professionals; and explaining one’s care preferences to a family member or friend (Sörensen et al., 2017). For individuals who manage chronic or progressive conditions, future care preparation may result in a future care plan that has been co-developed with their clinical team (Burt et al., 2014). However, engaging in preparation for future care needs can also occur prior to the onset of chronic or progressive conditions.

Adults’ early and consistent engagement in future care preparation offers a strong foundation for navigating aging-related health challenges. Indeed, older adults who plan for their future care have better health outcomes after transition into residential settings such as a residential aged care facility or a retirement village (Ball, 2005). For example, they tend to have a smoother psychological adjustment after transition into residential settings and to maintain autonomy and self-care for longer. Moreover, compared to those who do not engage in future care preparation, individuals who plan for their future care generally report less insecurity about the future (Sörensen & Pinquart, 2000), greater well-being (Noone et al., 2009), and lower depression and anxiety (Li et al., 2020; Silverstein et al., 2020; Sörensen et al., 2012a).

Lack of planning may instead lead to crisis decision-making among healthcare surrogates (Egan et al., 2023; Maloney et al., 1996) and can increase the risk of families making residential or care arrangements that are not well matched to an individual’s values and needs (Castle, 2003; George et al., 2019; Monagle, 2002; Silveira et al., 2010). This can consequently lead to diminished well-being and quality of life both to the individual and their family (Becker et al., 2023; Sörensen et al., 2012b). For example, after having taken a decision on behalf of the patient, healthcare surrogates can subsequently experience regret, self-blame, or doubt (Andersen et al., 2023).

Public health research has focused on future care preparation among people who have previously developed multiple chronic or progressive conditions or who are hospitalized (Lee et al., 2022; Morberg Jämterud & Sandgren, 2022). As such, the focus of research has primarily been on intervening by encouraging adults to prepare for likely, increasing dependence on others, rather than empowering them to understand and choose care options that are aligned with their values, whilst considering their family context and available resources. In this regard, advancing evidence on the predictors and correlates of future care preparation in the general population may inform future public health campaigns and/or strategies promoting future care preparation (Ahmed et al., 2020; Weathers et al., 2016). Promoting positive Views on Aging (VoA) may help with these goals.

### Views on Aging and Their Importance for Individuals’ Health and Future Care Preparation

VoA can be divided into General and Personal Views on Aging (GVoA and PVoA). While GVoA refer to the beliefs, ideas, and expectations individuals have about aging and older people in general, PVoA refer to individuals’ personal experiences of aging (Rothermund & Kornadt, 2015; Shrira et al., 2022). Individuals with negative GVoA may, for example, believe that aging is akin to bad health, memory loss, or disengagement, whereas individuals with positive GVoA may believe that older age is akin to wisdom, generativity, and psychological growth and aim to positively embrace their life. Individuals with negative PVoA, for example, perceive decreases in their energy and memory, whereas individuals with positive PVoA recognize positive aspects of aging such as having greater freedom (Miche et al., 2014). Although individuals’ GVoA and PVoA are often related, it may be possible, for example, that an individual in middle age has positive PVoA while holding negative GVoA. Hence, in research studies it is advisable to assess both GVoA and PVoA.

Empirical evidence suggests that in Western countries, the majority of middle-aged and older people have mostly negative VoA (World Health Organisation, 2021). The World Health Organization has considered this a problem because individuals with more negative VoA (comprising both GVoA and PVoA) typically have poorer health over time and a greater mortality risk (Chang et al., 2020; Westerhof et al., 2023). For example, in the United States, negative VoA are associated with 17.0 million cases of new illnesses each year, resulting in an annual cost of $63 billion (Levy et al., 2020). The influence of VoA on individuals’ health can be explained by Levy’s Stereotype Embodiment Theory (Levy, 2009). According to this theory, and supporting evidence, VoA influence health through three pathways: psychological, behavioral, and physiological. The psychological pathway suggests that more positive VoA may reinforce positive psychological characteristics such as self-efficacy, will to live, and a positive outlook toward the future (Dutt & Wahl, 2018; Wurm et al., 2013). The behavioral pathway reflects how VoA influence engagement in health-enhancing behaviors (e.g., engaging in physical activity and adhering to a healthy diet) and adaptive behaviors (e.g., seeking help and following medical recommendations when facing health issues) (Montepare, 2020; Sun & Smith, 2017). Lastly, the physiological pathway captures how negative VoA may lead to adverse biological processes (e.g., presence of biomarkers of inflammation such as C-reactive protein) and, consequently, to health-related conditions (Schönstein et al., 2022; Stephan et al., 2023).

Levy’s behavioral pathway may also explain how positive VoA can influence future care preparation (Kornadt et al., 2015; Levy, 2009). For example, it may be that individuals with positive VoA feel more in charge of their lives, their aging, and their care preparation options. The Health Belief Model may instead explain how negative VoA can influence future care preparation (Rosenstock, 1974). According to this model, individuals who have a disease or who experience significant age-related challenges and losses, and who perceive the given disease, loss, or challenge as harmful, are more likely to engage in future behaviors in relation to the given disease, loss, or challenge (Rosenstock, 1974). These future behaviors may include future care preparation. In support of this reasoning, COVID-19-related concerns (i.e., health-related worries) were found to be associated with greater aging preparation, a different but related concept (Kim-Knauss et al., 2022). Similarly, it may be that individuals with more negative general VoA (e.g., negative views of what they may experience in the future when older) engage more in future care preparation as an anticipatory adaptive behavior based on expected future age-related challenges (Brandtstädter & Rothermund, 2002; Lang, 2024).

Overall, VoA may be an understudied barrier or facilitator for future care preparation. Although existing evidence on this topic is scarce, one study found that more positive PVoA at baseline predicted subsequent increases in preparation for older age with regard to housing, work, social relations, and other domains in addition to health (Kornadt et al., 2015). To inform future work aiming to increase future care preparation among middle-aged and older adults, more research is needed to investigate associations of future care preparation not only in relation to PVoA but also in relation to GVoA. There is also the need to use measures of future care preparation that not only assess concrete steps (e.g., whether an individual has a care plan in place) but also capture the process of becoming aware of personal needs, gathering information about the support one may need, and decision making.

### The Current Study

This study aims to investigate whether PVoA (i.e., perceived age-related gains and losses) and GVoA (i.e., expectations regarding aging and (non) essentialist beliefs about aging) are cross-sectionally associated with future care preparation in a sample of community-dwelling individuals aged 50 and over and living in the United Kingdom. As VoA become increasingly self-relevant and important for health in older age (Kaspar et al., 2023), and older individuals are more likely to engage in future care preparation (Lang & Rupprecht, 2021; McGrew, 2000), the study also aims to test the moderating effect of age in these associations. As VoA are malleable (see Beyer et al., 2019; Knight et al., 2021; Zhu et al., 2024), collecting evidence on the potential association between VoA and future care preparation will suggest whether psychoeducational interventions and other types of strategies targeting VoA may also be useful to promote future care planning.

The study has the following hypotheses. First, it may be that those reporting higher perceived age-related gains also engage more in future care preparation. Second, it is hypothesized that those reporting higher perceived age-related losses will also report greater engagement in future care preparation. Third, it is expected that those with higher negative expectations regarding aging (especially in the physical health domain) engage more in future care preparation. Fourth, it is expected that those with more essentialist beliefs about aging (i.e., believing that aging is immutable and predetermined) will also report less future care preparation. Finally, we expect that the above associations are moderated by age.

## Method

We conducted an online cross-sectional survey using Qualtrics. We collected data from individuals aged 50 years and older; living in the United Kingdom; with a working email address and internet connection. We excluded participants who reported previously or currently providing care to an older person with progressive health conditions as caregivers may prepare differently for older age and care than non-caregivers (Kim-Knauss & Lang, 2021). Moreover, correlates of future care preparation for caregivers and non-caregivers have previously been investigated in a separate study by our team. Participants were recruited through advertisements posted on social media (i.e., X and LinkedIn) and in public places, Join Dementia Research, the Enabling Research in Care Homes **(**ENRICH) network, and snowball sampling. Potential participants who were interested in participating accessed the survey link via the study advert and provided their informed consent online. Participants were not paid to take part in the study but were told that £2500 was donated by the research team to a UK Charity. This study obtained governance and ethical approval from the Ethics and Governance Committee of the University of Surrey (EGA ref: FHMS 23-24 167 EGA).

### Measures

#### Future Care Preparation

This construct was assessed with a five-item questionnaire (Sörensen et al., 2017) that captures all aspects of future care preparation (i.e., awareness, information gathering, decision-making, and concrete steps). A sample item is *I have gathered information about options for care by talking to friends and/or relatives* (all items are reported in Supplemental Table 1). Each item is answered on a five-point scale (1 = *not at all true of me*; 2 = *not really true of me*; 3 = *neither true nor untrue*; 4 = *somewhat true of me*; 5 = *completely true of me*). The total score ranges from 5 to 25 with higher scores indicating greater future care preparation. In this sample Cronbach’s alpha for future care preparation was 0.71, indicating adequate internal reliability.

### Personal Views on Aging

#### Awareness of Age-Related Change

This construct was assessed with the ten-item version of the Awareness of Age-Related Change questionnaire (Kaspar et al., 2019). One of the benefits of this concept and questionnaire is that it individually captures both perceived age-related gains and losses. Indeed, it comprises five items assessing perceived age-related gains (i.e., *with my increasing age, I realize that I have more experience and knowledge to evaluate things and people*) and five items assessing perceived age-related losses (i.e., *with my increasing age, I realize that I have less energy*). Respondents rated how much each item applied to them on a five-point Likert scale (1 = *not at all*; 2 = *a little bit*; 3 = *moderately*; 4 = *quite a bit*; 5 = *very much*). Higher scores indicate higher perceived age-related gains and losses, respectively (range: 5–25). Hence, individuals can potentially score high both on perceived age-related gains and on perceived age-related losses. In this sample Cronbach’s alpha for perceived age-related gains was 0.73 and for perceived age-related losses was 0.79, indicating adequate internal reliability.

### General Views on Aging

#### Expectations Regarding Aging

This construct was assessed with the 12-item version of the Expectations Regarding Aging scale (Sarkisian et al., 2005). This scale comprises three subscales assessing the expectations people have for when they will be older or towards older people in the domains of mental health (e.g., *I expect that as I get older I will spend less time with friends and family*), physical health (e.g., *When people get older, they need to lower their expectations of how healthy they can be*), and cognitive function (e.g., *I expect that as I get older I will become more forgetful*). For each item, respondents choose an answer option from *definitely true* (1) to *definitely false* (4). For each subscale, the total score is obtained by summing the answer options for subscale items (range: 4–16). For all subscales, higher scores indicate more positive expectations regarding aging. Cronbach’s alphas for the expectations regarding aging in the mental health, physical health, and cognitive function subscales were 0.71, 0.77, and 0.82, respectively, indicating adequate to good internal reliability.

#### (Non) Essentialist Beliefs About Aging

This construct was assessed with a one-item question (Weiss & Diehl, 2021): *What do you think: Is the way we are aging immutable and predetermined by genetics or more malleable and changeable?* Participants provided a response by indicating where their perspective fell on a spectrum from *aging is genetically predetermined and immutable* (score of 0) to *aging is malleable and changeable* (score of 100).

#### Demographic Questions

These comprised age, sex, ethnic origin, highest level of education, marital status, and working status. Ethnic origin comprised *White* versus *Non-White* participants and was used solely for descriptive purposes, due to the very low number of *Non-White* participants. Highest level of education comprised the following options: primary education; secondary education; post-secondary education; vocational qualification; university degree. Marital status comprised the following answer options: married (including civil partnership); widowed; separated or divorced; co-habiting; and single. Working status comprised the following answer options: employed full-time; employed part-time; retired; unemployed.

#### Health

Participants were asked whether they had ever been diagnosed with a list of 23 physical health conditions, such as diabetes and stroke, and 19 mental health conditions, such as depression and anxiety (see Supplemental Text 1 for the full list of conditions). Two total scores were calculated for the number of physical and mental health conditions each participant had, with higher scores indicating a higher number of conditions.

### Data Analysis

We computed Pearson’s correlation coefficients for continuous variables and Spearman’s correlation coefficients for categorical variables. To investigate the cross-sectional associations between VoA indicators (independent variables) and future care preparation (dependent variable), we fitted unadjusted and adjusted linear regression models. In the adjusted models, we controlled for age, sex, education, marital status, working status, and number of physical and mental health conditions. These variables were chosen as covariates as they are related to PVoA, GVoA, and future care preparation (McGrew, 2000; Sabatini et al., 2020; Sabatini, Turner, et al., 2023; Su, 2008). The moderating effect of age in the associations of VoA indicators with future care preparation was tested by fitting interaction terms in the regression models. Unstandardized and standardized regression coefficients were reported. The effect size was indicated by standardized regression coefficients. Coefficients ≤0.09 were considered negligible, 0.10–0.29 small, 0.30–0.49 moderate, and ≥0.50 large (Cohen, 1988). Analyses were conducted using STATA version 17.

## Results

### Descriptive Statistics

The sample comprised 1,699 participants (mean age = 67.79 years; SD = 8.05 years; Table 1), of which 70.8% were women and 98.1% were white. Among participants, 55.5% obtained a university degree, 66.5% were married, and 73.9% were retired or unemployed. Participants had on average one physical health condition and no mental health conditions. Participants’ mean scores on perceived age-related gains and losses were 18.48 and 10.45, respectively, suggesting relatively high perceived age-related gains and few perceived age-related losses. Participants had more positive expectations regarding aging in the mental health domain (M = 12.06) compared to the physical health (M = 8.89) and cognitive function (M = 8.89) domains. Participants reported (non) essentialist beliefs about aging that translated to an average of 62.22/100, suggesting overall modest leaning towards believing that aging processes are malleable and controllable, rather than immutable. Out of a possible score of 25, participants scored 12.67 on future care preparation. Bivariate correlations among study variables are reported in Supplemental Table 2.

**Table 1.** Descriptive Statistics of Study Variables

Variables	Statistics
*N* =	1699
Age, M (SD)	67.79 (8.05)
Sex, *n* (%)	
Women	1201 (70.81)
Men	495 (29.19)
Ethnic origin, *n* (%)	
White	1661 (98.1)
Non-white	38 (1.9)
Education, *n* (%)	
No education, primary or secondary education	225 (13.4)
Post-secondary education	192 (11.4)
Vocational qualification	331 (19.7)
University degree	933 (55.5)
Marital status, *n* (%)	
Married or civil partnership	1118 (66.5)
Widowed	149 (8.9)
Divorced or separated	199 (11.8)
Co-habiting	87 (5.2)
Single	129 (7.7)
Working status, *n* (%)	
Employed full-time	271 (16.1)
Employed part-time	169 (10.0)
Retired or unemployed	1244 (73.9)
Number of physical health conditions, M (SD)	1.53 (0.95)
Number of mental health conditions, M (SD)	0.24 (0.66)
Perceived age-related gains, M (SD)	18.48 (3.77)
Perceived age-related losses, M (SD)	10.45 (3.37)
Expectations regarding aging—mental health, M (SD)	12.06 (2.38)
Expectations regarding aging—physical health, M (SD)	8.89 (2.39)
Expectations regarding aging—cognitive function, M (SD)	8.89 (2.39)
(Non) essentialists beliefs about aging, M (SD)	62.22 (19.25)

### Associations Between Perceived Age-Related Changes And Future Care Preparation

Results from unadjusted models are reported in Supplemental Text 2. In the adjusted linear regression model controlling for age, sex, education, marital status, working status, and number of physical and mental health conditions, higher perceived age-related gains (B = 0.12; 95% CI: 0.06, 0.17; β = 0.10; R^2^ = 0.02; Table 2; Supplemental Table 3) and higher perceived age-related losses (B = 0.16; 95% CI: 0.11, 0.22; β = 0.12, R^2^ = 0.01) were associated with greater future care preparation. All effects sizes were small according to Cohen (1988).

**Table 2.** Cross-Sectional Associations of Personal and General Views on Aging With Future Care Preparation—Adjusted Models

Independent variables	B (95% CI)	β	R^2^
Perceived age-related gains	0.12 (0.06; 0.17)***	0.10	0.02
Perceived age-related losses	0.16 (0.11; 0.22)***	0.12	0.01
Expectations regarding aging in the mental health domain	−0.07 (−0.16; 0.01)	−0.04	0.002
Expectations regarding aging in the physical health domain	−0.11 (−0.20; −0.03)**	−0.06	0.004
Expectations regarding aging in the cognitive function domain	−0.07 (−0.15; 0.02)	−0.04	0.002
Non-essentialists beliefs about aging	−0.004 (−0.01; 0.01)	−0.02	0.0004

B = unstandardized beta. CI = confidence interval. β = standardized beta. R^2^ = coefficient of determination. *p-Value <.05 **p-value <.01 ***p-value <.001. Models are adjusted for age, sex, education, marital status, working status, number of mental health conditions, and number of physical health conditions.

### Association Between Expectations Regarding Aging And Future Care Preparation

Results from unadjusted models are reported in Supplemental Text 2. In the adjusted linear regression model, more positive expectations regarding aging in the physical health domain were associated with less future care preparation (B = −0.11; 95% CI: −0.20, −0.03; β = −0.06; R^2^ = 0.004; Table 2; Supplemental Table 4), with a small effect size and modest variance in future care preparation explained. In the adjusted linear regression models, more positive expectations regarding aging in the mental health domain were not significantly associated with future care preparation. In the adjusted linear regression models, expectations regarding aging in the physical health domain were not significantly associated with future care preparation.

### Association Between (Non) Essentialist Beliefs About Aging and Future Care Preparation

(Non) Essentialists beliefs about aging were not significantly associated with future care preparation in the adjusted model (B = −0.004; 95% CI: −0.01; 0.01; β = −0.02; R^2^ = 0.0004; Table 2; Supplemental Table 5).

In all the above investigated models, among covariates, older age, being a woman, higher educational achievement, and more physical health conditions were associated with greater future care preparation (Supplemetal Tables 3–5).

### Moderating Effect of Age in the Associations of Views on Aging With Future Care Preparation

Age as a continuous variable did not moderate any of the associations of VoA indicators with future care preparation (p > 0.05).

## Discussion

This study used a large sample of individuals aged 50 years and older and living in the United Kingdom to investigate the cross-sectional associations of VoA with future care preparation, and the possible moderating role of age in these associations. Adjusted models showed that higher perceived age-related losses, less positive expectations regarding aging in the physical health domain, and higher perceived age-related gains were associated with greater future care preparation, that is, greater preparation for relying on others for support as new age-related challenges arise. All these associations were of small size. Finally, age did not moderate any of the investigated associations, suggesting that VoA may play a small role on preparation for future care irrespective of the participants’ age.

Regarding PVoA, this study found that higher perceived age-related gains and higher perceived age-related losses were associated with greater future care preparation. The finding that higher perceived age-related losses were associated with greater future care preparation may indicate that viewing one’s own aging as being dictated by losses increases fear for, or vigilance regarding, one’s health. This heightened fear or vigilance may, in turn, encourage individuals to equip themselves with information and plans that can help them mitigate expected challenges by relying on the care of others. To provide an example, if an individual is diagnosed with a progressive age-related condition (e.g., dementia and Parkinson’s disease) that involves an increasing loss of independence, being aware of one’s age-related challenges (negative PVoA) may be a prerequisite for engaging in preparation for future care. This reasoning would be in line with the Health Belief Model as individuals who experience age-related losses, and who interpret these losses as harmful, may be more likely to engage in future behaviors in relation to these losses, including future care preparation (Kim-Knauss et al., 2022; Rosenstock, 1974).

It is interesting that the current study found that those with more negative VoA may also be more likely to prepare for expected future negative changes. Indeed, individuals with more negative VoA tend to have worse health and greater risk of mortality over time (Chang et al., 2020; Levy et al., 2020; Westerhof et al., 2023) due to them being less proactive and less likely to adhere to healthy lifestyles (Klusmann et al., 2012; Sabatini et al., 2025). This lack of proactivity does not seem to be extended to future care preparation. It may be that, as individuals with negative VoA are more likely to experience anxiety and worry over time (Bodner et al., 2023; Freeman et al., 2016; Sabatini, Wahl, et al., 2023b), some levels of worry about the possibility of experiencing poor health and disability motivate them to engage in future care preparation (McMahan et al., 2020). For example, a study found that older adults with an elevated risk for Alzheimer’s disease became increasingly engaged in future care preparation behaviors such as awareness of needing help in the future, gathering information, decision making, and making concrete plans (Mak & Sörensen, 2012). Because the current study was cross-sectional, future longitudinal studies are needed to investigate the causal pathways among physical health, perceived losses, worry and fear for one’s health, and future care preparation.

Similar to results for perceived age-related losses, the current study found that less positive expectations regarding aging in the physical health domain—an indicator of GVoA—were related to greater engagement in preparation for future care needs. Negative GVoA are generally interpreted negatively by researchers due to individuals with more negative GVoA being at greater risk of poor health over time (World Health Organisation, 2021). In this case, more negative GVoA—a negatively biased depiction of older age—may make individuals concerned about ensuring they have support from others as they age. Consequently, individuals with more negative GVoA may be more prone to take action and anticipatorily plan for their future care (Lang, 2024).

It is however important to acknowledge that if people have mostly negative VoA, they may have completely defeated, helpless attitudes or may be too anxious about aging to do anything productive to plan (Mroz et al., 2024). Indeed, our pattern of results suggests that individuals may not only need to experience some age-related losses but also be somewhat positive about their own aging, to engage in future care preparation. In line with our finding, in a previous study, more positive PVoA at baseline predicted subsequent increases in preparation for physical health changes (Kornadt et al., 2015). It may be that individuals with more positive PVoA feel more in charge of their lives, including decisions about their future care. This would be aligned with the behavioral pathway postulated in Levy’s Stereotype Embodiment Theory (Levy, 2009). Indeed, compared to those with more negative PVoA, individuals with more positive PVoA are generally more careful about their own health and they follow a healthy lifestyle (Klusmann et al., 2019). This proactive behavior of individuals with positive PVoA may extend to future care preparation (Klusmann et al., 2012, 2019).

Overall, our results suggest a relationship between VoA and future care preparation. As VoA are malleable, psychoeducational and behavioral interventions that promote positive PVoA (see Beyer et al., 2019; Knight et al., 2021; Zhu et al., 2024) may be useful to also elicit future care preparation. More specifically, as this study found that both more negative VoA and more positive PVoA are associated with greater future care preparation, future psychoeducational interventions targeting VoA to promote greater future care preparation should strive for a delicate balance between acknowledging the challenges that old age can involve while maintaining a positive outlook by reminding participants also of the positive aspects of aging. So far, psychoeducational interventions promoting positive VoA have been delivered via face-to-face sessions to small groups of participants (Zhu et al., 2024). The strength of in-person psychoeducational sessions is that they allow small groups of older adults to interactively reflect on and share their age-related experiences and views. Future policies should foster/support the implementation of such interventions, for example, through the institution of community-based aging workshops. However, future efforts could also be directed towards making these interventions more accessible to the wider population. For instance, psychoeducational sessions could be shortened in a way that they could be delivered during primary care visits, or they could be digitalized to reach also individuals who live in more remote areas. At the same time, study results suggest that the discussions that healthcare professionals typically have with patients with regard future care preparation could start from an initial discussion of patients’ personal and general VoA as this preliminary conversation may augment their motivation to engage in future care preparation.

### Strengths and Limitations

Strengths of this study are the inclusion of measures assessing both PVoA and GVoA, as they assess two different facets of VoA which may not be associated with the same correlates, and the large sample size. This study also has several limitations. A first limitation of this study is its cross-sectional nature, which did not allow investigation of the causal pathways of VoA with future care preparation. A second limitation is that participants completed the survey online so the study sample excluded individuals who do not have access to the internet. Third, the participants had above average levels of education with 55.5% who had obtained a university degree (UK Parliament). Hence, the sample may not necessarily be representative of the general UK population. Fourth, this study used a brief five-item measure to assess future care preparation which did not allow investigation of individual aspects of preparation (e.g., awareness, information gathering, and decision making) in relation to VoA. Fifth, the associations identified in this study between VoA and future care preparation were of small size. This may be due to the study sample having reported generally low perceived age-related losses. It may be that these associations are stronger in samples of individuals who have perceived more age-related changes. Indeed, in this study we found an association between higher perceived age-related losses and greater future care preparation. Levels of future care preparation were also low in this study sample. Finally, this study focused on the role of age as possible moderator in the association of VoA with future care preparation. Other variables not analyzed in this study, such as frailty and self-rated health, should be tested as possible moderators.

## Conclusions

The current study suggests that VoA may be associated with future care preparation. That is, those people who are more aware of both the positive and negative age-related changes they experience and who have more negative expectations with regard to the physical health of older people are more likely to prepare more, mentally and practically, for the reality that they are likely to rely on others for support with daily functioning as they age. Interventions promoting positive VoA would therefore not only promote more accurate VoA and greater (self-)inclusion of older people but may also foster future care preparation. Finally, given the possible associations between how individuals view aging and future care preparation, encouraging older adults to have open and frank conversations about their VoA with family, friends, and/or healthcare professionals may be an important first step in eliciting care needs.

## Supplemental Material

Supplemental Material - Cross-Sectional Associations Between Personal and General Views on Aging and Future Care Preparation in Individuals Aged 50 and Over and Living in the United Kingdom Supplemental Material for Cross-Sectional Associations Between Personal and General Views on Aging and Future Care Preparation in Individuals Aged 50 and Over and Living in the United Kingdom by Serena Sabatini, Fiona S. Rupprecht, Emily Mroz, Shelbie Turner, Robyn Capithorne Crainey, Eugene Y. H. Tang, Claire V. Burley, Jennifer Dunne, Elissa Burton, Stephanie Wong, Bethany Wilton-Harding, Tim Windsor, Rachel C. Ambagtsheer, Katya Numbers, and Blossom Stephan in Journal of Applied Gerontology.

## Data Availability

Study data will be deposited in Open Science Framework and made freely accessible in April 2026.*
