# Supplementary material for: Cross-Sectional Associations Between Personal and General Views on Aging and Future Care Preparation in Individuals Aged 50 and Over and Living in the United Kingdom
Source: J Appl Gerontol. 2025 Nov 20;45(11):2235–46. doi: 10.1177/07334648251398098 (PMC13672912; doi:10.1177/07334648251398098)
Supplement: Supplementary file 1 [file sj-pdf-1-jag-10.1177_07334648251398098.pdf]

Cross-Sectional Associations Between Personal and General Views on Aging and Future Care Preparation in Individuals Aged 50 and Over and Living in the United Kingdom

Supplementary Table 1.
Descriptive Statistics for Preparation for Future Care Items

Item	Not at all true of me	Not really true of me	Neither true nor untrue	Somewhat true of me	Completely true of me
			N (%)		
I have thought about whether I might need help or care in the future.	171 (10.1)	284 (16.8)	334 (19.7)	716 (42.2)	191 (11.3)
I try not to think about things like future loss of independence.	257 (15.2)	582 (34.3)	402 (23.7)	337 (19.9)	117 (6.9)
I have gathered information about options for care by talking to friends and/or relatives.	893 (52.6)	293 (17.3)	242 (14.3)	226 (13.3)	43 (2.5)
If I ever need help or care, I can choose between several options that I have considered in some depth.	695 (41.0)	381 (22.5)	324 (19.1)	242 (14.3)	55 (3.2)
I have explained to someone close to me what my care preferences are	655 (38.6)	249 (14.7)	159 (9.4)	390 (23.0)	245 (14.4)

Supplementary Table 2.
Correlations Among Study Variables.

	1.	2.	3.	4.	5.	6.	7.	8.	9.	10.	11.	12.	13.
1. Age	__												
2. Sex	**-0.09; .0002**	__											
3. Education	0.01; .749	**-0.07; .002**	__										
4. Marital status	0.01; .697	**0.13; .001**	-0.02; .442	__									

5. Working status	**0.46; .001**	0.001; .974	-0.001; .984	**-0.05; .028**	__								
6. Number of mental health conditions	**-0.18; .001**	0.05; .059	**-0.08; .002**	**0.10; .0001**	-0.04; .075	__							
7. Number of physical health conditions	**0.19; .001**	-0.03; .227	-0.03; .179	**0.05; .048**	**0.15; .001**	**0.14; .001**	__						
8. Perceived age-related gains	**-0.07; .003**	**0.11; .001**	-0.03; .238	-0.01; .844	0.01; .837	**-0.05; .049**	-0.03; .293	__					
9. Perceived age-related losses	**0.18; .001**	**-0.06; .009**	**-0.08; .002**	0.03; .263	**0.15; .001**	**0.21; .001**	**0.25; .001**	-0.04; .141	__				
10. Expectations regarding aging - physical health	**-0.11; .001**	**0.18; .001**	**0.06; .013**	**0.08; .001**	**-0.05; .025**	-0.02; .481	**-0.10; .0001**	**0.08; .002**	**-0.43; .001**	__			
11. Expectations regarding aging - mental health	0.01; .809	**0.05; .038**	**0.16; .001**	-0.03; .202	0.01; .566	**-0.12; .001**	**-0.07; .005**	**0.13; .001**	**-0.36; .001**	**0.41; .001**	__		
12. Expectations regarding aging - cognitive function	**-0.08; .001**	**0.12; .001**	**0.08; .002**	**0.07; .006**	**-0.06; .023**	0.03; .293	-0.05; .061	**0.09; .0002**	**-0.37; .001**	**0.53; .001**	**0.36; .001**	__	
13.	**0.0**	0.31	0.04	**0.0**	0.00	-	-	**0.1**	**-**	**0.1**	**0.1**	**0.1**	__

(Non) essentialists beliefs about aging	**6; .021**	; .196	; .084	**5; .042**	2; .936	0.05; .062	0.01; .761	**3; .001**	**0.13; .001**	**7; .001**	**2; .001**	**0; .0001**	
14. Future care preparation	**0.16; .001**	**0.09; .0002**	**0.07; .004**	-0.01; .781	**0.10; .001**	-0.03; .267	**0.09; .0002**	**0.11; .001**	**0.13; .001**	**-0.06; .016**	-0.02; .405	-0.03; .194	-0.01; .813

Note: significant correlations are highlighted in bold.

Supplementary Table 3.
Cross-Sectional Associations of Perceived Age-Related Gains and Losses With Future Care Preparation – Adjusted Models

	Perceived Age-Related Gains as Independent Variable			Perceived Age-Related Losses as Independent Variable		
Independent variables	B (95% CI)	β	R^2	B (95% CI)	β	R^2
Perceived age-related gains/losses	0.12 (0.06; 0.17)***	0.10	0.02	0.16 (0.11; 0.22)***	0.12	0.01
Age	0.07 (0.04; 0.10)***	0.14	0.01	0.06 (0.02; 0.09)***	0.10	0.01
Sex	1.03 (0.58; 1.48)***	0.11	0.01	1.16 (0.71; 1.61)***	0.12	0.01
Education						
Primary or secondary education	-0.96 (-1.57; -0.36)**	-0.08	0.01	-1.05 (-1.65; -0.44)***	-0.08	0.01
Post-secondary education	-0.89 (-1.53; -0.24)**	-0.07	0.004	-0.99 (-1.65; -0.35)**	-0.08	0.01
Vocational qualification	-0.29 (-0.81; 0.24)	-0.03	0.001	-0.25 (-0.77; 0.27)	-0.02	0.001
University degree	Reference category			Reference category		
Marital status						
Married or civil partnership	Reference category			Reference category		
Widowed	0.43 (-0.31; 1.16)	0.03	0.001	0.42 (-0.31; 1.16)	0.03	0.001
Divorced or separated	0.03 (-0.61; 0.67)	0.002	0.00	0.09 (-0.55; 0.72)	0.01	0.00
Co-habiting	-0.64 (-1.55; 0.27)	-0.03	0.001	-0.62 (-1.54; 0.30)	-0.03	0.001
Single	-0.33 (-1.12; 0.44)	-0.02	0.0004	-0.42 (-1.20; 0.36)	-0.03	0.001
Working status						

Employed full-time	-0.05 (-0.68; 0.58)	-0.004	0.00	-0.15 (-0.78; 0.48)	-0.01	0.0001
Employed part-time	-0.13 (-0.85; 0.59)	-0.01	0.0001	-0.09 (-0.81; 0.63)	-0.01	0.00
Retired or unemployed	Reference category	Reference category		Reference category	Reference category	
Number of mental health conditions	-0.06 (-0.37; 0.26)	-0.01	0.0001	-0.25 (-0.57; 0.07)	-0.04	0.001
Number of physical health conditions	0.30 (0.08; 0.52)**	0.07	0.004	0.20 (-0.02; 0.42)	0.04	0.002

B= Unstandardized beta. CI= Confidence intervals. β= Standardized beta. R^2= Coefficient of Determination. * p-value <.05 ** p-value < .01 *** p-value <.001

Supplementary Table 4.

Cross-Sectional Association Between Expectations Regarding Aging and Future Care Preparation – Adjusted Models

	Expectations regarding aging in the mental health domain as independent variable			Expectations regarding aging in the physical health domain as independent variable			Expectations regarding aging in the cognitive function domain as independent variable		
Independent variables	B (95% CI)	β	R^2	B (95% CI)	β	R^2	B (95% CI)	β	R^2
Expectations regarding aging	-0.07 (-0.16; 0.01)	-0.04	0.002	-0.11 (-0.20; -0.03)**	-0.06	0.004	-0.07 (-0.15; 0.02)	-0.04	0.002
Age	0.06 (0.03; 0.10)***	0.12	0.01	0.06 (0.03; 0.09)***	0.12	0.01	0.06 (0.03; 0.09)***	0.12	0.01
Sex	1.13 (0.68; 1.58)***	0.12	0.01	1.21 (0.75; 1.66)***	0.13	0.02	1.15 (0.70; 1.60)***	0.12	0.01
Education									
Primary or secondary education	-1.03 (-1.65; -0.42)***	-0.08	0.01	-1.01 (-1.61; -0.40)***	-0.08	0.01	-0.99 (-1.60; -0.39)***	-0.08	0.01
Post-secondary education	-0.94 (-1.60; -0.29)**	-0.07	0.01	-0.92 (-1.57; -0.27)**	-0.07	0.004	-0.91 (-1.56; -0.26)**	-0.07	0.004
Vocational qualification	-0.28 (-0.81; 0.25)	-0.03	0.001	-0.26 (-0.79; 0.26)	-0.02	0.001	-0.25 (-0.78; 0.28)	-0.02	0.001
University degree	Reference category			Reference category			Reference category		
Marital status									
Married or civil partnership	Reference category			Reference category			Reference category		
Widowed	0.49 (-0.25; 1.23)	0.03	0.001	0.55 (-0.19; 1.29)	0.04	0.001	0.53 (-0.21; 1.27)	0.04	0.001
Divorced or separated	0.08 (-0.56; 0.72)	0.01	0.00	0.12 (-0.51; 0.76)	0.01	0.0001	0.10 (-0.54; 0.74)	0.01	0.0001
Co-habiting	-0.64 (-1.56; 0.28)	-0.03	0.001	-0.61 (-1.53; .31)	-0.03	0.001	-0.63 (-1.55; 0.29)	-0.03	0.001
Single	-0.42 (-1.20; 0.36)	-0.03	0.001	-0.38 (-1.16; 0.40)	-0.02	0.001	-0.40 (-1.18; 0.39)	-0.02	0.001
Working status									

Employed full-time	-0.20 (-0.83; 0.44)	-0.02	0.0002	-0.19 (-0.82; 0.45)	-0.02	0.0002	-0.18 (-0.81; 0.46)	-0.02	0.0002
Employed part-time	-0.18 (-0.90; 0.54)	-0.10	0.0001	-0.15 (-0.87; -0.57)	-0.01	0.0001	-0.18 (-0.89; 0.54)	-0.01	0.0001
Retired or unemployed	Reference category			Reference category			Reference category		
Number of mental health conditions	-0.13 (-0.44; 0.19)	-0.02	0.0004	-0.11 (-0.42; 0.21)	-0.02	0.0003	-0.10 (-0.41; 0.46)	-0.02	0.0002
Number of physical health conditions	0.29 (0.07; 0.51)*	0.06	0.004	0.27 (0.06; 0.49)*	0.06	0.004	0.29 (0.07; 0.51)**	0.07	0.004

B= Unstandardized beta. CI= Confidence intervals. β= Standardized beta. R^2= Coefficient of Determination. * p-value <.05 ** p-value < .01 *** p-value <.001

Supplementary Table 5.

Cross-Sectional Association Between (Non) Essentialists Beliefs About Aging and Future Care Preparation – Adjusted Model

	Adjusted model		
Independent variables	B (95% CI)	β	R^2
Non essentialists beliefs about aging	-0.004 (-0.01; 0.01)	-0.02	0.0004
Age	0.06 (0.03; 0.09)***	0.12	0.01
Sex	1.10 (0.65; 1.56)***	0.12	0.01
Education			
Primary or secondary education	-0.93 (-1.55; -0.31)**	-0.07	0.01
Post-secondary education	-0.87 (-1.53; -0.21)**	-0.07	0.004
Vocational qualification	-0.24 (-0.78; 0.30)	-0.02	0.001
University degree	Reference category	Reference category	
Marital status			
Married or civil partnership	Reference category	Reference category	
Widowed	0.56 (-0.19; 1.31)	0.04	0.001
Divorced or separated	-0.03 (-0.68; 0.63)	-0.001	0.00
Co-habiting	-0.82 (-1.77; 0.13)	-0.04	0.002
Single	-0.49 (-1.28; 0.30)	-0.03	0.001
Working status			
Employed full-time	-0.12 (-0.77; 0.52)	-0.01	0.0001
Employed part-time	-0.20 (-0.93; 0.53)	-0.01	0.0002
Retired or unemployed	Reference category	Reference category	
Number of mental health conditions	-0.14 (-0.48; 0.19)	-0.02	0.0004
Number of physical health conditions	0.28 (0.05; 0.50)*	0.06	0.003

B= Unstandardized beta. CI= Confidence intervals. β= Standardized beta. R^2= Coefficient of Determination. * p-value <.05 ** p-value < .01 *** p-value <.001

Supplementary Text One.

Physical Health. Participants were asked they had ever been diagnosed with one of the following physical health conditions: high blood pressure; stroke; heath disease/ heart attack/ angina; diabetes; mild cognitive impairment; Parkinson's disease; high cholesterol; hypothyroidism; hyperthyroidism; arthritic condition; Huntington's disease; current cancer; cancer in full remission; osteoporosis; asthma; epilepsy; motor neurone disease; multiple sclerosis; Paget's disease; deep vein thrombosis; human immunodeficiency virus; acquired immunodeficiency disease syndrome; and hepatitis C virus.

Mental Health. Participants were asked whether they had ever been diagnosed with one of the following mental health conditions: depression; mania, hypomania, bipolar or manic-depression; anxiety, nerves or generalized anxiety disorder; social anxiety or social phobia; agoraphobia; panic attacks; obsessive compulsive order; anorexia nervosa; bulimia nervosa; psychological over-eating or binge-eating; schizophrenia; any other type of psychosis or psychotic illness; a personality disorder; autism, Aspergers or autistic spectrum disorder; attention deficit or attention deficit and hyperactivity disorder.

Supplementary Text Two.

Results from univariable linear regression models.

Associations Between Perceived Age-Related Changes And Future Care Preparation

In the unadjusted linear regression models, higher perceived age-related gains (B= 0.12; 95% CI: 0.07, 0.17; p-value= .001; β= 0.11; R^2= 0.01; Table 2) and higher perceived age-related

losses (B= 0.16; 95% CI: 0.11, 0.22; p-value< .001; β= 0.14; R^2= 0.02; Table 2) were associated with greater future care preparation.

Association Between Expectations Regarding Aging And Future Care Preparation

In the unadjusted (B= -0.10; 95% CI: -0.19, -0.02; β= -0.06; R^2=0.003; Table 3) linear regression models, more positive expectations regarding aging in the physical health domain were associated with less future care preparation, with a smalleffect size and modest variance in future care preparation explained. In the unadjusted linear regression models, more positive expectations regarding aging in the mental health domain were not significantly associated with future care preparation (B= -0.04; 95% CI: -0.12; 0.05; β= -0.02; R^2= 0.0004; Table 3). In the unadjusted linear regression models (Table 6) expectations regarding aging in the cognitive function domain were not significantly associated with future care preparation (B= -0.06; 95% CI: -0.14; 0.03; β= -0.03; R^2= 0.001).

Association Between (Non) Essentialist Beliefs About Aging And Future Care Preparation

(Non) Essentialists beliefs about aging were not significantly associated with future care preparation in the unadjusted models (B= -0.001; 95% CI: -0.01; 0.01; β= -0.01; R^2= 0.00; Table 7).
